# Current treatment paradigm and survival outcomes among patients with newly diagnosed multiple myeloma in China: a retrospective multicenter study

**DOI:** 10.20892/j.issn.2095-3941.2022.0612

**Published:** 2023-01-12

**Authors:** Huishou Fan, Weida Wang, Ya Zhang, Jianxiang Wang, Tao Cheng, Lugui Qiu, Xin Wang, Zhongjun Xia, Gang An

**Affiliations:** 1 State Key Laboratory of Experimental Hematology, National Clinical Research Center for Blood Diseases, Haihe Laboratory of Cell Ecosystem, Institute of Hematology & Blood Diseases Hospital, Chinese Academy of Medical Sciences & Peking Union Medical College, Tianjin 300020, China; 2 State Key Laboratory of Oncology in South China, Collaborative Innovation Center for Cancer Medicine, Sun Yat-Sen University Cancer Center, Guangzhou 510060, China; 3 Department of Hematology, Shandong Provincial Hospital Affiliated to Shandong First Medical University, Jinan 250021, China

**Keywords:** Multiple myeloma, autologous stem cell transplantation, minimal residual disease, survival outcomes, multicenter study

## Abstract

**Objective:**

Evidence on the prognostic value of autologous stem cell transplantation (ASCT) and minimal residual disease (MRD) dynamics of patients with newly diagnosed multiple myeloma (NDMM) in China is limited. Our objective in the current study was to understand the current care paradigm and outcomes of these patients.

**Methods:**

This longitudinal cohort study used historical data from three top-tier hematologic disease care hospitals that contributed to the National Longitudinal Cohort of Hematological Diseases-Multiple Myeloma. Treatment regimens [proteasome inhibitor (PI)-, immunomodulatory drug (IMiD)-, PI+IMiD-based, and conventional], post-induction response, ASCT and MRD status, and survival outcomes [progression-free survival (PFS) and overall survival (OS)] were evaluated.

**Results:**

In total, 454 patients with NDMM were included (median age, 57 years; 59.0% males) with a median follow-up of 58.7 months. The overall response rate was 91.0%, 83.9%, 90.6%, and 60.9% for PI-, IMiD-, PI+IMiD-based, and conventional regimens, respectively. Patients with ASCT during first-line therapy (26.2%) had a longer PFS and OS than patients who did not receive ASCT [median PFS, 42.9 *vs.* 21.2 months, *P* < 0.001; median OS, not reached (NR) *vs*. 65.8 months, *P* < 0.001]. The median OS was NR, 71.5, and 56.6 months among patients with sustained MRD negativity, loss of MRD negativity, and persistent MRD, respectively (*P* < 0.001). Multivariate analysis revealed that the lactic dehydrogenase level, International Staging System stage, extra-medullary disease, and upfront ASCT were independent factors in predicting OS among NDMM patients.

**Conclusions:**

Our study showed that novel agent-based regimens, first-line ASCT, and sustained MRD negativity were associated with a superior outcome for patients with NDMM in China (Identifier: NCT04645199).

## Introduction

Multiple myeloma (MM) is the second most prevalent hematologic malignancy^1,2^. As the most populous country, China has the second highest number of incident cases of MM worldwide, with an age-standardized incidence rate (95% uncertainty interval) of 1.03 [0.88–1.17] per 100,000 persons recorded in 2016, corresponding to approximately 16,500 new cases^3^. As such, MM is a significant contributor to the burden of disease in China, as well as globally^4^.

Over recent decades, with the wide clinical application of novel agents and autologous stem cell transplantation (ASCT), the prognosis of patients with MM has been remarkably improved^5–7^. Indeed, results from China have suggested that mortality among patients with MM remained stable in recent years, despite the continuous rise in disease incidence^3^. Nevertheless, delivery of care for patients with MM is often hindered by a lack of access to advanced medical practice and heterogeneity in MM with respect to patient characteristics and risk stratification, thus leading to dismal outcomes among patients in developing countries, including China^3,8^. According to the CONCORD-3 2018 statistics, the overall 5-year survival rate of MM is 24.8% in China, which is still significantly lower than the 46.7% in the United States and other countries in Asia, such as 33.3% in Japan^9^.

Even though a small number of patients with MM can be considered “functionally” cured^10^, approximately 15% have a median survival < 2 years^11^. Thus, individualized therapy, close monitoring, and treatment modification continue to be critical in improving patient outcomes^12^. Clinical evidence has shown that assessment of minimal residual disease (MRD) predicts disease prognosis and helps guide treatment decisions^13^. Furthermore, a diversity in MRD features has been observed in diagnostic high- and low-risk MM^14^; however, there is limited real-world evidence to date on the prognostic value of ASCT and MRD dynamics in Chinese patients with newly diagnosed multiple myeloma (NDMM).

To that end, we conducted this study to understand the current care paradigm and outcomes of patients with NDMM in China based on a multicenter, longitudinal cohort study. This information could provide the necessary evidence to guide modification of individualized therapies and improve patient outcomes.

## Material and methods

### Data source

Data collected through the National Longitudinal Cohort of Hematological Diseases (NICHE) were analyzed in this study. The NICHE study is an ongoing, longitudinal, national, multi-disease cohort evaluation of patients with hematologic diseases^15^. The NICHE study was established in 2016 by the Institute of Hematology and Blood Diseases Hospital (IHBDH)^15^. This study utilized the NICHE-MM sub-cohort, with historical, anonymized electronic and paper medical records (01/01/2013–02/17/2022) and follow-up data (including records of clinical visits and regular telephone follow-ups that occurred during the follow-up period). Three hospitals for hematologic disease care have contributed to the NICHE-MM study: the IHBDH; the Shandong Provincial Hospital; and the Sun Yat-Sen University Cancer Center (SYSUCC). Data on patient demographics, disease characteristics, treatment regimens, treatment response, and survival were evaluated for this study, which was reviewed and approved by the Institutional Review Board of all participating hospitals before the study initiation. The NICHE-MM study was reviewed and approved by the Human Genetic Resource Administration of China.

### Study design and population

An overview of the study design is shown in **Supplementary Figure S1**. This retrospective, longitudinal cohort study used data systematically captured by hospital information systems. A total of 957 patients with NDMM diagnosed between 01/01/2013–12/31/2017 (the enrollment period) were reviewed from the NICHE-MM study. We excluded 364 patients who were diagnosed at the study site, but treated at other hospitals (*n* = 105), and patients with inadequate information in the electronic medical record (*n* = 259). Of the remaining 593 patients, 139 were further excluded because of their participation in clinical trials (*n* = 112) or because of other primary malignancies at the time of diagnosis (*n* = 27). Finally, 454 patients with NDMM who underwent consecutive treatments (≥ 4 cycles of induction therapy) were included in this study (**Supplementary Figure S2**). The follow-up period for each patient was defined as the interval between the index date and the end of data availability (i.e., date of last follow-up or death, whichever occurred earlier). The last wave of telephone follow-ups was conducted before 02/17/2022 to ascertain survival status. Loss to follow-up was defined as the inability to trace the patients > 3 times for the scheduled hospital or telephone visits during the follow-up period. Mortality data were collected for each patient, with confirmed date and cause of death.

### Treatment options and criteria for response

Treatment regimens for induction therapy included proteasome inhibitor (PI)-, immunomodulatory drug (IMiD)-, PI+IMiD-based, and conventional regimens (**Supplementary Table S1**). Response outcomes were assessed by physicians according to the International Myeloma Working Group uniform response criteria^12^: the overall response rate (ORR) was calculated as the sum of the stringent complete response; complete response (CR); very good partial response (VGPR); and partial response (PR).

### iFISH and MRD detection

Purified CD138^+^ plasma cells, followed by interphase fluorescence *in situ* hybridization (iFISH), were performed as previously reported^16^. The iFISH panel included deletion (del)(13q14), del(17p13), 1q21 gain/amplification (1q21+), translocation (t)(11;14) (q13;q32), t(4;14) (p16.3;q32), and t(14;16) (q32;q23).The threshold levels were defined as 20% for deletion or amplification and 10% for translocation. High-risk cytogenetic abnormality (HRCA) was defined as the presence of t(4;14), t(14;16), or del(17p13)^17^. Standard-risk cytogenetic abnormality (SRCA) was defined as absence of these abnormalities^17^. Multiparameter flow cytometry (MFC), using two combinations of 8-color monoclonal antibodies, was performed at the IHBDH and SYSUCC for MRD assessment (at a sensitivity level of 10^−5^–10^−4^). The cut-off for MRD negativity was set at < 50 clonal plasma cells of > 500,000 nucleated cells (10^−4^). The regular flow-MRD examination was initially assessed in patients with a ≥ VGPR post-induction therapy, then performed after ASCT/consolidation treatment, and approximately every 6 months during maintenance and at the time of progression. MRD dynamics were also used to stratify patients for comparison of survival outcomes according to post-induction therapy and follow-up monitoring. Sustained MRD negativity was defined as MRD negativity lasting ≥ 12 months.

### Statistical analysis

Survival outcomes included progression-free survival (PFS), which was defined as the time period from the start of treatment to disease progression, death, or the last follow-up evaluation^12^, and overall survival (OS) was defined as the time period from the start of treatment to any-cause death or the last follow-up evaluation. PFS and OS were estimated using the Kaplan–Meier method, with differences between and among subgroups assessed using the log-rank test. When comparing ≥ 3 groups, if the overall difference was statistically significant, the Bonferroni method was used to correct the significance level and conduct a pairwise comparison. The median PFS (mPFS) and median OS (mOS) with 95% confidence intervals (CIs) and the *P*-values of log-rank tests were reported. Variants with *P*-values < 0.1 in the univariate Cox analysis were included in the multivariate Cox analysis. Considering the important impact of age on OS, we included age groups in the multivariable model. Multivariate Cox proportional hazards regression models were fitted to analyze the risk factors associated with OS. No violation of the proportional hazards assumption was observed according to the Schoenfeld residuals test. Hazard ratios (HRs) with 95% CIs and two-sided *P*-values were reported. All analyses were conducted in R (version 4.1.2).

## Results

### Characteristics of the study population

A total of 454 patients were enrolled in the study, including 267 from the IHBDH, 138 from the SYSUCC, and 49 from the Shandong Provincial Hospital (**Supplementary Figure S3**). The median [interquartile range (IQR)] of follow-up for the study population throughout the study period was 58.7 [45.8–75.7] months (**Supplementary Figure S3**), and the median [IQR] number of follow-up visits throughout the study period was 45 [30–79.5]. By the end of the follow-up period, 4.2% of the study population were lost to follow-up and 165 (36.3%) patients had died.

The median [range] age of the overall population at the time of diagnosis was 57 [26, 81] years and 59.0% were male. In total, 119 (26.2%) of the 454 patients received ASCT during the first-line of therapy, and the median [range] age was younger among patients who received ASCT treatment compared to patients who did not receive ASCT (50 [32, 67] *vs*. 57 [26, 81], *P* < 0.001). Other details of the characteristics of the study population are shown in **Table 1**.

**Table 1:** Patient demographics and disease characteristics of patients with MM

Characteristics	No. of patients (%)			*P* value
	Non-ASCT	ASCT	Total	
Median age [range], years	58 [26, 81]	50 [32, 67]	57 [26, 81]	< 0.001
Age group at diagnosis, years				< 0.001
≤ 65	275 (82.1)	115 (96.6)	390 (85.9)	
> 65	60 (17.9)	4 (3.4)	64 (14.1)	
Gender				0.302
Female	142 (42.4)	44 (37.0)	186 (41.0)	
Male	193 (57.6)	75 (63.0)	268 (59.0)	
M-component				0.155
IgG	155 (46.3)	54 (45.4)	209 (46.0)	
IgA	76 (22.7)	21 (17.6)	97 (21.4)	
IgD	18 (5.4)	14 (11.8)	32 (7.0)	
Light chain	76 (22.7)	25 (21.0)	101 (22.2)	
Other	10 (3.0)	5 (4.2)	15 (3.3)	
LDH				0.118
Normal	273 (81.5)	89 (74.8)	362 (79.7)	
Elevated	62 (18.5)	30 (25.2)	92 (20.3)	
ISS stage				0.009
I	63 (19.1)	36 (31.0)	99 (22.2)	
II	120 (36.4)	44 (37.9)	164 (36.8)	
III	147 (44.5)	36 (31.0)	183 (41.0)	
R-ISS stage				< 0.001
I	7 (3.5)	14 (16.9)	21 (7.4)	
II	156 (77.2)	56 (67.5)	212 (74.4)	
III	39 (19.3)	13 (15.7)	52 (18.2)	
Extra-medullary disease				0.780
Yes	63 (18.8)	21 (17.6)	84 (18.5)	
No	272 (81.2)	98 (82.4)	370 (81.5)	
Induction regimen				
PI-based	203 (60.6)	88 (73.9)	291 (64.1)	0.050
IMiD-based	52 (15.5)	10 (8.4)	62 (13.7)	
PI+IMiD-based	19 (5.7)	13 (10.9)	32 (7.0)	
Conventional	61 (18.2)	8 (6.7)	69 (15.2)	
lq21+				0.759
Positive	66 (51.6)	29 (49.2)	95 (50.8)	
Negative	62 (48.4)	30 (50.8)	92 (49.2)	
Del(17p13)				0.496
Positive	12 (9.4)	4 (6.5)	16 (8.4)	
Negative	116 (90.6)	58 (93.5)	174 (91.6)	
Del(13q14)				0.246
Positive	64 (50.0)	25 (41.0)	89 (47.1)	
Negative	64 (50.0)	36 (59.0)	100 (52.9)	
t(4;14)				0.056
Positive	26 (35.1)	7 (17.9)	33 (29.2)	
Negative	48 (64.9)	32 (82.1)	80 (70.8)	
t(11;14)				0.141
Positive	20 (27.4)	16 (41.0)	36 (32.1)	
Negative	53 (72.6)	23 (59.0)	76 (67.9)	
t(14;16)				0.645
Positive	4 (5.5)	3 (7.7)	7 (6.2)	
Negative	69 (94.5)	36 (92.3)	105 (93.8)	
Cytogenetics^†^				0.415
High risk	36 (30.0)	14 (24.1)	50 (28.1)	
Standard risk	84 (70.0)	44 (75.9)	128 (71.9)	

ASCT, autologous stem cell transplantation; IgH, immunoglobulin heavy chain; IMiD, immunomodulatory drug; LDH, lactate dehydrogenase; MM, multiple myeloma; PI, proteasome inhibitor; ISS, International Staging System; R-ISS, Revised-International Staging System. ^†^High-risk cytogenetics was defined by the presence of t(4;14), t(14;16), or del(17p13). Standard risk cytogenetics was defined as the absence of these abnormalities.

### Post-induction responses and survival outcomes among patients undergoing different treatment regimens

Among the 453 patients with post-induction responses information, the ORRs were 91.0%, 83.9%, 90.6%, and 60.9% for patients who received PI-, IMiD-, PI+IMiD-based, and conventional regimens, respectively. Specifically, the proportion of patients who achieved a ≥ CR was 35.9%, 19.4%, 28.1%, and 4.3%, respectively (**Supplementary Figure S4**). The mOS was 89.3 [95% CI: 71.4, not reached (NR)], 69.3 (95% CI: 59.3, NR), NR (95% CI: 44.7, NR), and 53.8 (95% CI: 39.3, NR) months for patients who were treated with PI-, IMiD-, PI+IMiD-based, and conventional regimens, respectively. Although the overall difference across the four groups was only marginally statistically significant (*P* = 0.050; **Supplementary Figure S5**), patients treated with PI-based regimens demonstrated a higher survival probability than patients who received conventional treatments (Bonferroni adjusted *P* = 0.029).

### Survival outcomes for patients who did and did not receive first-line ASCT

**Figure 1:**
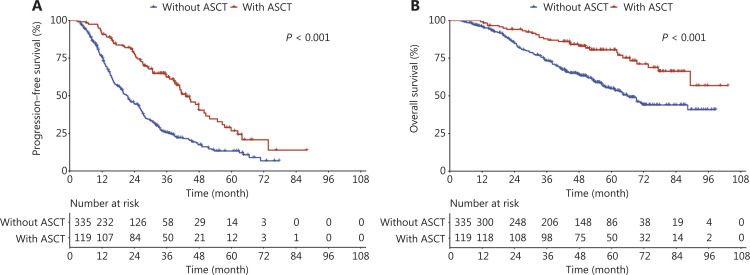
Survival outcomes between patients who received first-line ASCT and patients who did not receive first-line ASCT. (A) Progression-free survival by ASCT status, (B) overall survival by ASCT status.

Disease progression occurred in 65 (54.6%) of the patients who received ASCT and 246 (73.4%) of the patients who did not receive ASCT. The mPFS was 42.9 (95% CI: 38.9, 50.5) and 21.2 (95% CI: 18.8, 25.1) months among patients who received ASCT compared to patients who did not receive ASCT, respectively (*P* < 0.001). The mOS was NR and 65.8 (95% CI: 57.8, NR) among patients who received ASCT compared to patients who did not receive ASCT, respectively (*P* < 0.001; **Figure 1**). Even among patients with Revised-International Staging System (R-ISS) II/III disease, ASCT was shown to significantly improve survival for both PFS and OS (**Supplementary Figure S6**).

### Post-ASCT responses further distinguished the prognosis among patients who received ASCT

**Figure 2:**
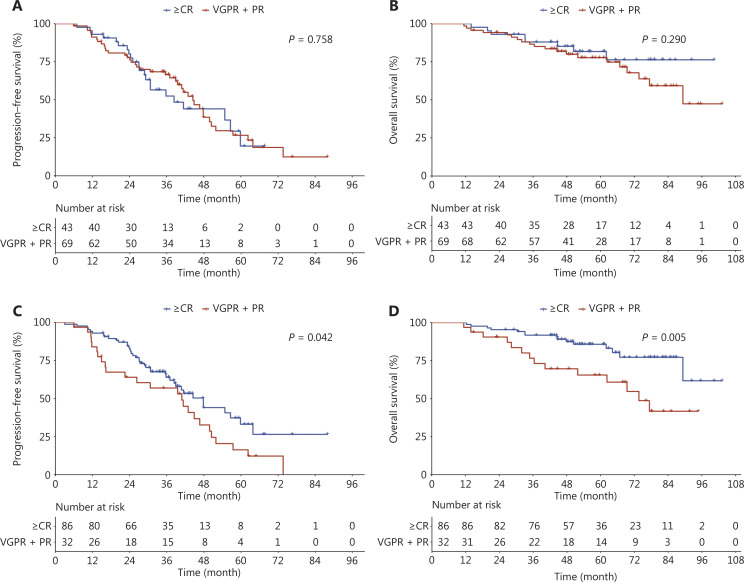
Survival outcomes according to pre- and post-ASCT responses among patients with first-line ASCT. (A) Progression-free survival and (B) overall survival according to patient pre-ASCT responses, and (C) progression-free survival and (D) overall survival according to patient post-ASCT responses.

Among patients who received ASCT, PFS and OS did not differ significantly between patients who achieved ≥ CR (*n* = 43) and patients who only achieved VGPR or PR (*n* = 69) according to the pre-ASCT responses. The mPFS was 38.4 (95% CI: 29.2, NR) months *vs*. 44.5 (95% CI: 39.5, 51.9) months, respectively (*P* = 0.758; **Figure 2A**), and the mOS was NR *vs*. 89.3 (95% CI: 77.4, NR) months, respectively (*P* = 0.290; **Figure 2B**).

According to the post-ASCT responses, patients who achieved ≥ CR (*n* = 86) had a longer PFS (*P* = 0.042) and OS (*P* = 0.005) compared with patients who only achieved VGPR or PR (*n* = 32). The mPFS was 47.8 (95% CI: 38.4, 63.9) months *vs*. 40.9 (95% CI: 22.5, 50.5) months, respectively (**Figure 2C**), and the mOS was NR *vs*. 73.7 (95% CI: 62.3, NR) months, respectively (**Figure 2D**).

### Survival outcomes among patients with MRD dynamics

**Figure 3:**
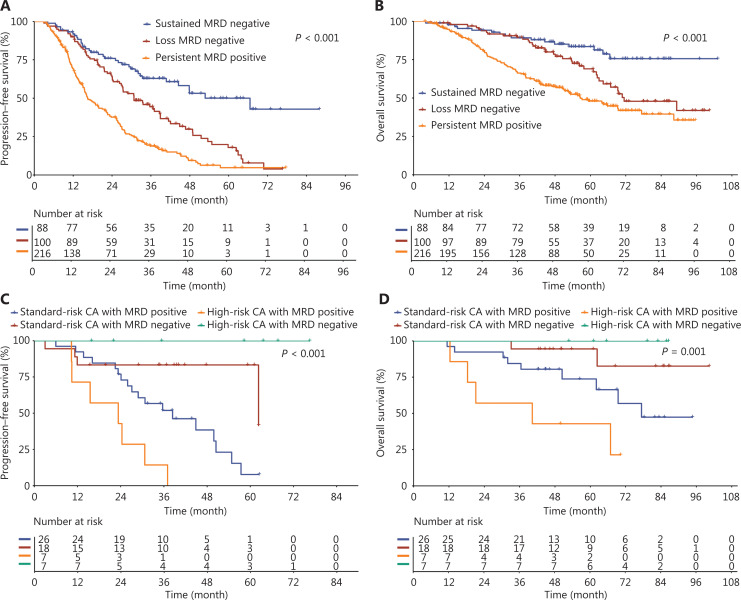
Survival outcomes according to MRD dynamics or final MRD status combined with genetic risk stratifications (A) Progression-free survival and (B) overall survival by MRD dynamics. (C) Progression-free survival and (D) overall survival by final MRD status in combination with different genetic risk stratifications.

Among the 404 patients who received regular MRD monitoring, 88 had sustained MRD negativity, 100 had loss of MRD negativity, and 216 had persistent MRD positivity. The mPFS was 52.7 (95% CI: 44.5, NR), 30.8 (95% CI: 26.8, 38.9), and 16.4 (95% CI: 15.4, 21.6) months among patients with sustained MRD negativity, loss of MRD negativity, and persistent MRD positivity, respectively (*P* < 0.001; **Figure 3A**). The mOS was NR, 71.5 (95% CI: 64.3, NR), and 56.6 (95% CI: 50.4, 77.4) months among patients with sustained MRD negativity, loss of MRD negativity, and persistent MRD positivity, respectively (*P* < 0.001; **Figure 3B**).

### Final MRD negativity may overcome the dismal prognostic impact of HRCA

Among the 14 patients with HRCA, mPFS was NR for those with MRD negativity and 23.3 (95% CI: 10.4, NR) months among those with MRD positivity (Bonferroni adjusted *P* = 0.004; **Figure 3C**). Similarly, the mOS was NR for those with MRD negativity and 40.3 (95% CI: 18.3, NR) months among those with MRD positivity (Bonferroni adjusted *P* = 0.045; **Figure 3D**). Among the 44 patients with SRCA, patients with MRD negativity also had a longer PFS (Bonferroni adjusted *P* = 0.060) and OS (Bonferroni adjusted *P* = 0.443) than patients with MRD positivity, although the differences were not statistically significant (**Figure 3C and 3D**).

### Prognostic predictors for OS

A multivariable analysis consisting of age groups and the established survival risk factors by univariate analysis [including elevated lactate dehydrogenases (LDH), extra-medullary disease, clinical stage, cytogenetics, and ASCT; **Table 2**] revealed that an elevated LDH level, ISS III disease, and extra-medullary disease predicts significantly poor survival, while upfront ASCT appeared to be an independent protective factor for OS.

**Table 2:** Univariate and multivariate models on overall survival in patients with NDMM

Variable	Univariate analysis		Multivariate analysis	
	HR (95% CI)	*P* value	HR (95% CI)	Adjusted *P* value
Age, years				
> 65 *vs*. ≤ 65	1.11 (0.71, 1.73)	0.6	1.04 (0.65, 1.66)	0.9
Elevated LDH				
Yes *vs*. No	2.21 (1.55, 3.16)	< 0.001	2.38 (1.65, 3.44)	< 0.001
Extramedullary disease				
Yes *vs*. No	1.66 (1.15, 2.38)	0.006	1.74 (1.19, 2.53)	0.004
ISS at diagnosis				
II *vs*. I	0.99 (0.63, 1.55)	> 0.9	1.01 (0.64, 1.60)	> 0.9
III *vs*. I	1.64 (1.08, 2.50)	0.020	1.57 (1.01, 2.43)	0.044
High risk cytogenetics^†^				
Yes *vs*. No	1.55 (0.94, 2.56)	0.084	1.53 (0.92, 2.55)	0.10
Upfront ASCT				
Yes *vs*. No	0.45 (0.30, 0.67)	< 0.001	0.46 (0.30, 0.70)	< 0.001

ASCT, autologous stem cell transplantation; LDH, lactate dehydrogenase; NDMM, newly diagnosed multiple myeloma; ISS, International Staging System. ^†^High-risk cytogenetics was defined by the presence of t(4;14), t(14;16), or del(17p13).

## Discussion

This retrospective study was conducted to examine the current care paradigm and associated clinical outcomes among Chinese patients with NDMM based on data from the NICHE-MM cohort. The NICHE-MM multicenter cohort collects data from three hematologic disease care hospitals located in northern, eastern, and southern China, and thus provides reasonable geographic coverage for the Chinese population of patients with NDMM. Additionally, data collection from the NICHE-MM multicenter cohort followed a standardized protocol across study centers to improve the data validity, with regular follow-up evaluations collected until February 2022 (a median follow-up time of 58.7 months of the NICHE-MM cohort and a high frequency of follow-up visits at a median number of 45 visits) to supplement survival status ascertainment, thereby providing an up-to-date, comprehensive assessment of patients from China using longer follow-up data compared with previous studies^18–20^.

In agreement with previous studies^21–23^, our study showed a greater treatment response in patients with novel agents treatment (e.g., PI-, PI+IMiD-, or IMiD-based regimens) compared to patients who received conventional therapy. In addition, ASCT is recommended for transplant-eligible NDMM patients for survival benefits, even among patients with HRCA^24–28^. Similarly, patients in our study population who received ASCT also had better survival than patients who did not receive ASCT, and such benefits remained significant among patients with R-ISS II/III disease. With a median follow-up time of 58.7 months, the PFS and OS for our study population who did and did not receive ASCT were slightly lower compared with the results from prior clinical trial observations with a similar duration of follow-up^25^. In agreement with a previous study^29^, patients who achieved ≥ CR after ASCT demonstrated better survival than patients who only achieved VGPR or PR after ASCT. Moreover, we also provided supportive evidence indicating that it was more important to achieve deep remission after ASCT than before ASCT.

Only 26.2% of our study population received ASCT, despite previous research showed a continuously increasing trend of ASCT use in China^18–20,30,31^. The ASCT rate in China remains significantly lower than developed countries (76% in the Australian and New Zealand registry data)^32^. Possible explanations for this finding might be the low ASCT technology penetration rate, and patient financial constraints, although China has included the cost of ASCT as part of the overall treatment of MM in the scope of medical insurance reimbursement. Considering that ASCT is a relatively cost-effective treatment option^33^ and confers survival benefits for patients with NDMM, the implementation of ASCT promotion and education programs to improve the awareness of ASCT in transplantation-eligible patients remain important in the future.

As one of the first multicenter studies to evaluate the prognostic value of MRD dynamics for patients with MM in China, our findings demonstrated the clinical importance of MRD evaluation by showing that patients with sustained MRD negativity had both superior PFS and OS, compared with patients with persistent MRD positivity and those with loss of MRD negativity, which aligns with the results of studies from other countries^34–36^. Although the survival outcomes for MM are improving in the era of novel treatments, patients with HRCA have worse outcomes compared to patients with SRCA^37,38^. A limited number of studies, mostly conducted outside China, have previously explored the prognosis of NDMM according to MRD dynamics and cytogenetic-based risk stratification^39^.Results in our study showed that PFS and OS were better in patients with HRCA who were MRD negative than in patients who were MRD positive, indicating that achieving MRD negativity may overcome the poor prognostic impact of HRCA. Therefore, for patients with HRCA, achieving MRD negativity may be the optimal treatment endpoint^40^. Additionally, multivariate Cox regression analysis in our study suggested that an elevated LDH level, ISS III disease, and extra-medullary disease may be poor prognostic predictors, while upfront ASCT appeared to be an independent protective factor in NDMM patients.

This study had limitations. First, a small proportion (4.2%) of patients were lost to follow-up, which may have contributed to potential selection bias. Second, as the survival status for some patients was ascertained through telephone follow-up, measurement errors and misclassifications of mortality may exist. Third, a limited sample size was available in each subgroup in the analyses comparing PFS and OS by final MRD status with different CA risk categories. Fourth, although data from a multicenter cohort was included, the findings may only reflect the survival of MM patients receiving consecutive treatment in China’s advanced MM care centers. Therefore, the findings might not be generalizable to the overall Chinese population of patients with NDMM. Finally, it may be that because only 14.1% of patients were > 65 years in this study (*n* = 64), the age group (> 65 *vs*. ≤ 65 years) did not show independent prognostic ability in multivariate Cox regression analysis. Therefore, further investigation in prognostic value of age groups is warranted. Like most other studies, a limited number of patients with cytogenetic detection were included and the study may therefore have been underpowered to define the prognostic value of HRCA. As these are preliminary findings from the NICHE-MM cohort, further research is warranted to expand our analyses and examine the prognostic value of the important risk factors identified in this population.

## Conclusions

In conclusion, we provided a contemporary characterization of Chinese patients with NDMM and the current care pathways, treatment responses, and survival outcomes. The clear benefits of novel agent-based treatment, upfront ASCT, and sustained MRD negativity on survival outcomes provided supportive evidence of the clinical utility in managing Chinese patients with NDMM.

## References

[1] Raab MS, Podar K, Breitkreutz I, Richardson PG, Anderson KC (2009). Multiple myeloma. Lancet.

[2] Palumbo A, Anderson K (2011). Multiple myeloma. N Engl J Med.

[3] Liu J, Liu W, Mi L, Zeng X, Cai C, Ma J (2019). Incidence and mortality of multiple myeloma in China, 2006-2016: an analysis of the Global Burden of Disease Study 2016. J Hematol Oncol.

[4] Cowan AJ, Allen C, Barac A, Basaleem H, Bensenor I, Curado MP (2018). Global burden of multiple myeloma: a systematic analysis for the Global Burden of Disease Study 2016. JAMA Oncol.

[5] Sant M, Minicozzi P, Mounier M, Anderson LA, Brenner H, Holleczek B (2014). Survival for haematological malignancies in Europe between 1997 and 2008 by region and age: results of EUROCARE-5, a population-based study. Lancet Oncol.

[6] Engelhardt M, Terpos E, Kleber M, Gay F, Wäsch R, Morgan G (2014). European Myeloma Network recommendations on the evaluation and treatment of newly diagnosed patients with multiple myeloma. Haematologica.

[7] Blimark CH, Turesson I, Genell A, Ahlberg L, Björkstrand B, Carlson K (2018). Outcome and survival of myeloma patients diagnosed 2008-2015. Real-world data on 4904 patients from the Swedish Myeloma Registry. Haematologica.

[8] de Mel S, Lim SH, Tung ML, Chng WJ (2014). Implications of heterogeneity in multiple myeloma. Biomed Res Int.

[9] Allemani C, Matsuda T, Di Carlo V, Harewood R, Matz M, Niksic M (2018). Global surveillance of trends in cancer survival 2000-14 (CONCORD-3): analysis of individual records for 37 513 025 patients diagnosed with one of 18 cancers from 322 population-based registries in 71 countries. Lancet.

[10] Barlogie B, Mitchell A, van Rhee F, Epstein J, Morgan GJ, Crowley J (2014). Curing myeloma at last: defining criteria and providing the evidence. Blood.

[11] Pawlyn C, Morgan GJ (2017). Evolutionary biology of high-risk multiple myeloma. Nat Rev Cancer.

[12] Durie BG, Harousseau JL, Miguel JS, Bladé J, Barlogie B, Anderson K (2006). International uniform response criteria for multiple myeloma. Leukemia.

[13] Kumar S, Paiva B, Anderson KC, Durie B, Landgren O, Moreau P (2016). International Myeloma Working Group consensus criteria for response and minimal residual disease assessment in multiple myeloma. Lancet Oncol.

[14] Ding H, Xu J, Lin Z, Huang J, Wang F, Yang Y (2021). Minimal residual disease in multiple myeloma: current status. Biomark Res.

[15] Clinicaltrials.gov National Longitudinal Cohort of Hematological Diseases[EB/OL].

[16] An G, Yan Y, Xu Y, Mao X, Liu J, Fan H (2020). Monitoring the cytogenetic architecture of minimal residual plasma cells indicates therapy-induced clonal selection in multiple myeloma. Leukemia.

[17] Palumbo A, Avet-Loiseau H, Oliva S, Lokhorst HM, Goldschmidt H, Rosinol L (2015). Revised international staging system for multiple myeloma: a report from International Myeloma Working Group. J Clin Oncol.

[18] He J, Yang L, Han X, Zheng G, Zheng W, Wei G (2014). The choice of regimens based on bortezomib for patients with newly diagnosed multiple myeloma. PLoS One.

[19] Lu J, Lu J, Chen W, Huo Y, Huang X, Hou J (2014). Clinical features and treatment outcome in newly diagnosed Chinese patients with multiple myeloma: results of a multicenter analysis. Blood Cancer J.

[20] Geng C, Liu N, Yang G, Liu A, Leng Y, Wang H (2013). Retrospective analysis of 264 multiple myeloma patients. Oncol Lett.

[21] Goldschmidt H, Ashcroft J, Szabo Z, Garderet L (2019). Navigating the treatment landscape in multiple myeloma: which combinations to use and when?. Ann Hematol.

[22] Moreau P, Richardson PG, Cavo M, Orlowski RZ, San Miguel JF, Palumbo A (2012). Proteasome inhibitors in multiple myeloma: 10 years later. Blood.

[23] Gandolfi S, Laubach JP, Hideshima T, Chauhan D, Anderson KC, Richardson PG (2017). The proteasome and proteasome inhibitors in multiple myeloma. Cancer Metastasis Rev.

[24] Al Hamed R, Bazarbachi AH, Malard F, Harousseau JL, Mohty M (2019). Current status of autologous stem cell transplantation for multiple myeloma. Blood Cancer J.

[25] Morè S, Corvatta L, Manieri VM, Saraceni F, Scortechini I, Mancini G (2022). Autologous stem cell transplantation in multiple myeloma: where are we and where do we want to go?. Cells.

[26] Harousseau JL, Moreau P (2009). Autologous hematopoietic stem-cell transplantation for multiple myeloma. N Engl J Med.

[27] Mikhael J, Ismaila N, Cheung MC, Costello C, Dhodapkar MV, Kumar S (2019). Treatment of multiple myeloma: ASCO and CCO Joint Clinical Practice Guideline. J Clin Oncol.

[28] Dimopoulos MA, Moreau P, Terpos E, Mateos MV, Zweegman S, Cook G (2021). Multiple myeloma: EHA-ESMO Clinical Practice Guidelines for diagnosis, treatment and follow-up. Ann Oncol.

[29] van de Velde H, Londhe A, Ataman O, Johns HL, Hill S, Landers E (2017). Association between complete response and outcomes in transplant-eligible myeloma patients in the era of novel agents. Eur J Haematol.

[30] Xu LP, Lu PH, Wu DP, Sun ZM, Liu QF, Han MZ (2021). Hematopoietic stem cell transplantation activity in China 2019: a report from the Chinese Blood and Marrow Transplantation Registry Group. Bone Marrow Transplant.

[31] Xu LP, Wu DP, Han MZ, Huang H, Liu QF, Liu DH (2017). A review of hematopoietic cell transplantation in China: data and trends during 2008-2016. Bone Marrow Transplant.

[32] Bergin K, Wellard C, Augustson B, Cooke R, Blacklock H, Harrison SJ (2021). Real-world utilisation of ASCT in multiple myeloma (MM): a report from the Australian and New Zealand myeloma and related diseases registry (MRDR). Bone Marrow Transplant.

[33] Pandya C, Hashmi S, Khera N, Gertz MA, Dispenzieri A, Hogan W (2014). Cost-effectiveness analysis of early vs. late autologous stem cell transplantation in multiple myeloma. Clin Transplant.

[34] San-Miguel J, Avet-Loiseau H, Paiva B, Kumar S, Dimopoulos MA, Facon T (2022). Sustained minimal residual disease negativity in newly diagnosed multiple myeloma and the impact of daratumumab in MAIA and ALCYONE. Blood.

[35] Bianchi G (2022). Sustained minimal residual disease in myeloma. Blood.

[36] Avet-Loiseau H, San-Miguel J, Casneuf T, Iida S, Lonial S, Usmani SZ (2021). Evaluation of sustained minimal residual disease negativity with daratumumab-combination regimens in relapsed and/or refractory multiple myeloma: analysis of POLLUX and CASTOR. J Clin Oncol.

[37] Avet-Loiseau H, Attal M, Campion L, Caillot D, Hulin C, Marit G (2012). Long-term analysis of the IFM 99 trials for myeloma: cytogenetic abnormalities [t(4;14), del(17p), 1q gains] play a major role in defining long-term survival. J Clin Oncol.

[38] Avet-Loiseau H, Attal M, Moreau P, Charbonnel C, Garban F, Hulin C (2007). Genetic abnormalities and survival in multiple myeloma: the experience of the Intergroupe Francophone du Myélome. Blood.

[39] Munshi NC, Avet-Loiseau H, Anderson KC, Neri P, Paiva B, Samur M (2020). A large meta-analysis establishes the role of MRD negativity in long-term survival outcomes in patients with multiple myeloma. Blood Adv.

[40] Li H, Li F, Zhou X, Mei J, Song P, An Z (2019). Achieving minimal residual disease-negative by multiparameter flow cytometry may ameliorate a poor prognosis in MM patients with high-risk cytogenetics: a retrospective single-center analysis. Ann Hematol.

