# Supplementary material for: Current treatment paradigm and survival outcomes among patients with newly diagnosed multiple myeloma in China: a retrospective multicenter study
Source: Cancer Biol Med. 2023 Jan 12;20(1):77–87. doi: 10.20892/j.issn.2095-3941.2022.0612 (PMC9843447; doi:10.20892/j.issn.2095-3941.2022.0612)
Supplement: Supplementary file 1 [file cbm-20-077-s001.pdf]

Supplementary materials

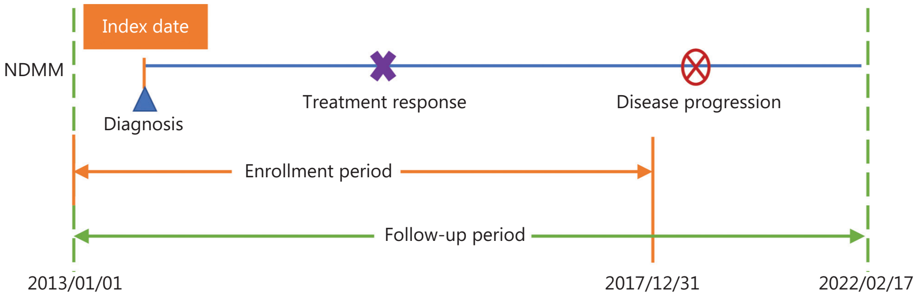

Figure S1 Overview of the study design. NDMM, newly diagnosed multiple myeloma.

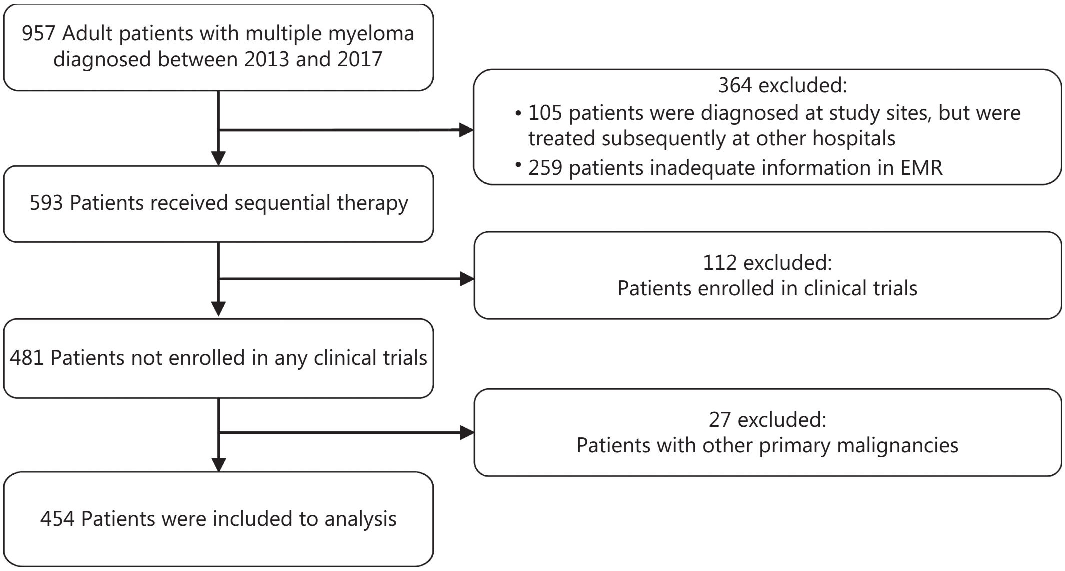

Figure S2 Study population. EMR, electronic medical records.

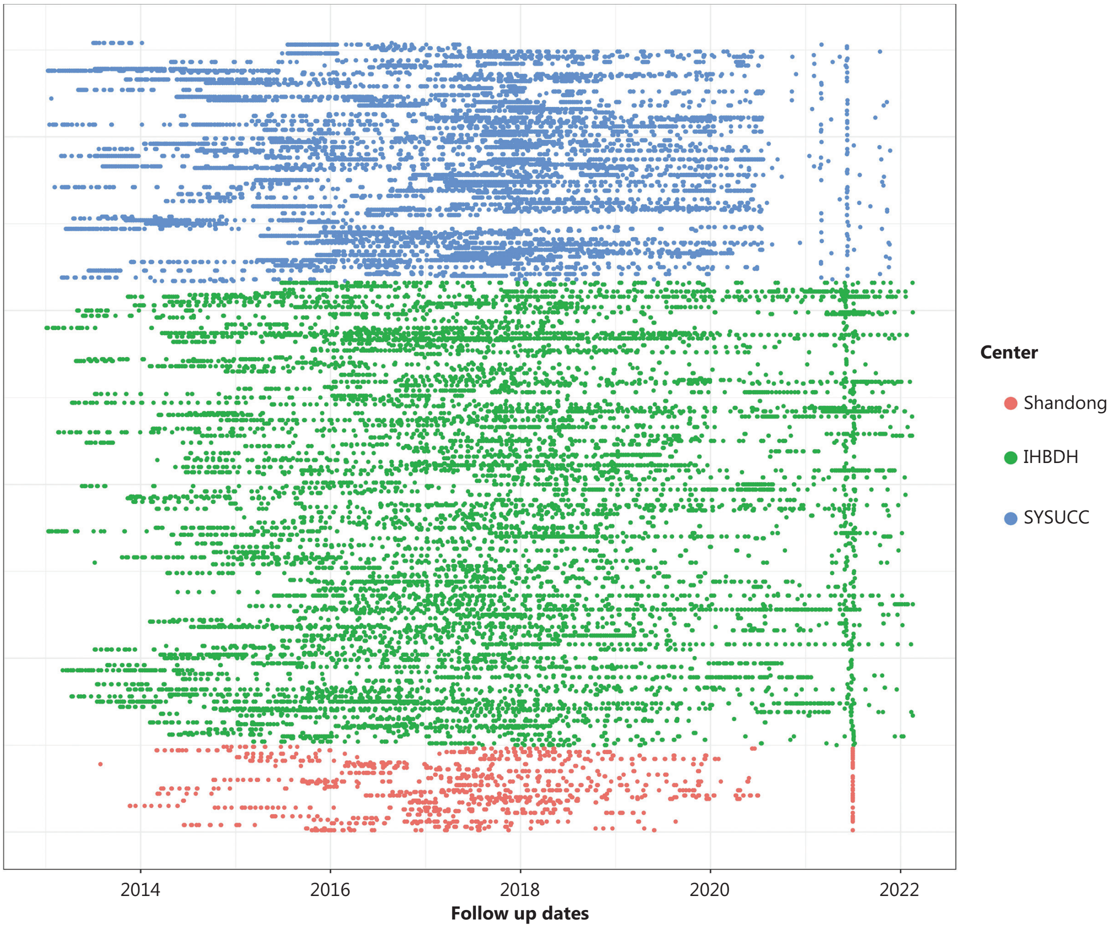

Figure S3 Spaghetti plot of follow-up time for patients. IHBDH, Institute of Hematology and Blood Diseases Hospital; Shandong, Shandong Provincial Hospital; SYSUCC, Sun Yat-Sen University Cancer Center.

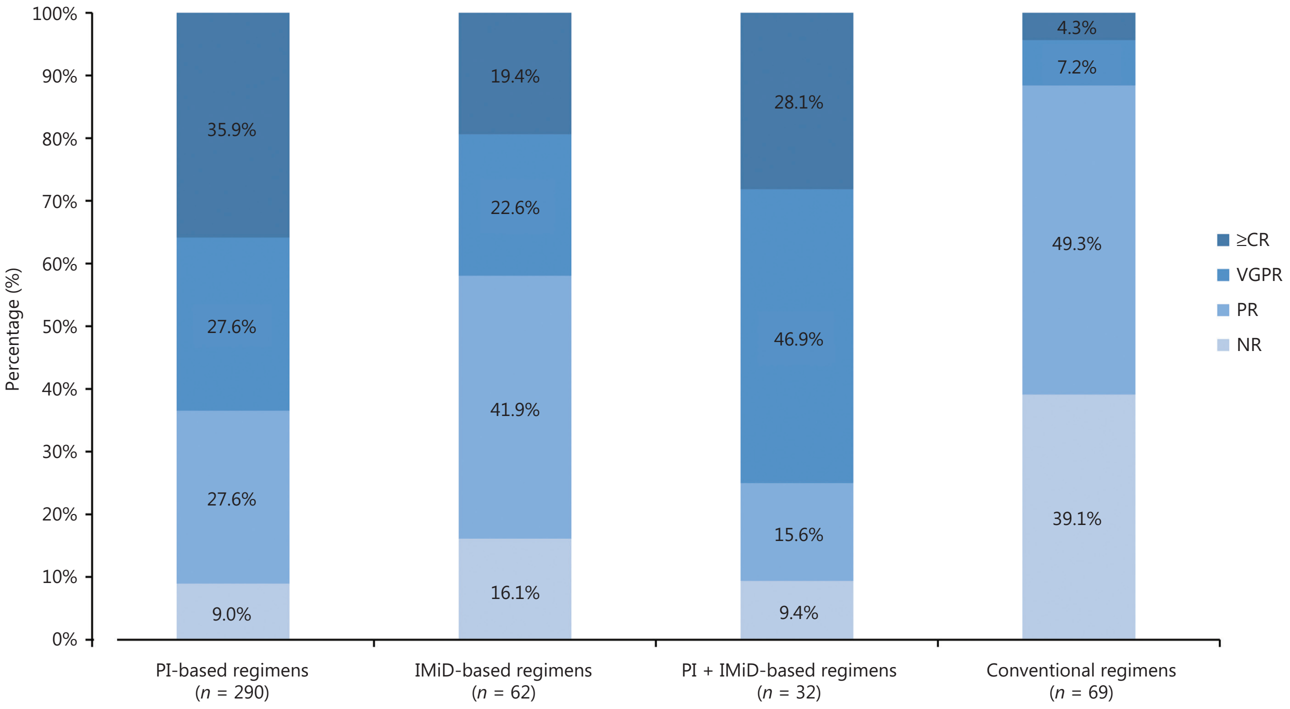

Figure S4 Induction treatment response among patients with different treatment regimens. CR, complete response; IMiD, immunomodulatory drugs; NR, no response; PI, proteasome inhibitor; PR, partial response; and VGPR, very good partial response.

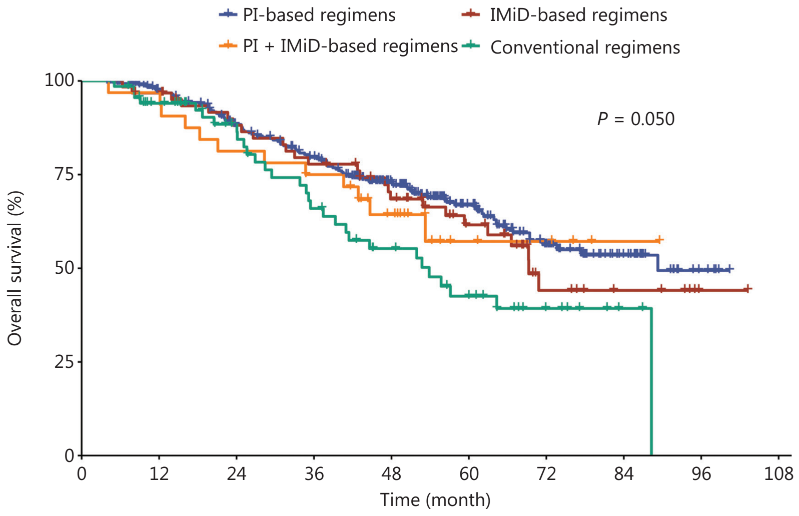

Number at risk

	0	12	24	36	48	60	72	84	96	108
PI-based regimens	291	270	235	204	149	91	49	24	5	0
IMiD-based regimens	62	59	51	45	36	24	10	6	1	0
PI + IMiD-based regimens	32	31	26	23	15	5	4	1	0	0
Conventional regimens	69	58	44	32	23	16	7	2	0	0

Time (month)

Figure S5 Overall survival among patients with different treatment regimens. IMiD, immunomodulatory drug; PI, proteasome inhibitor.

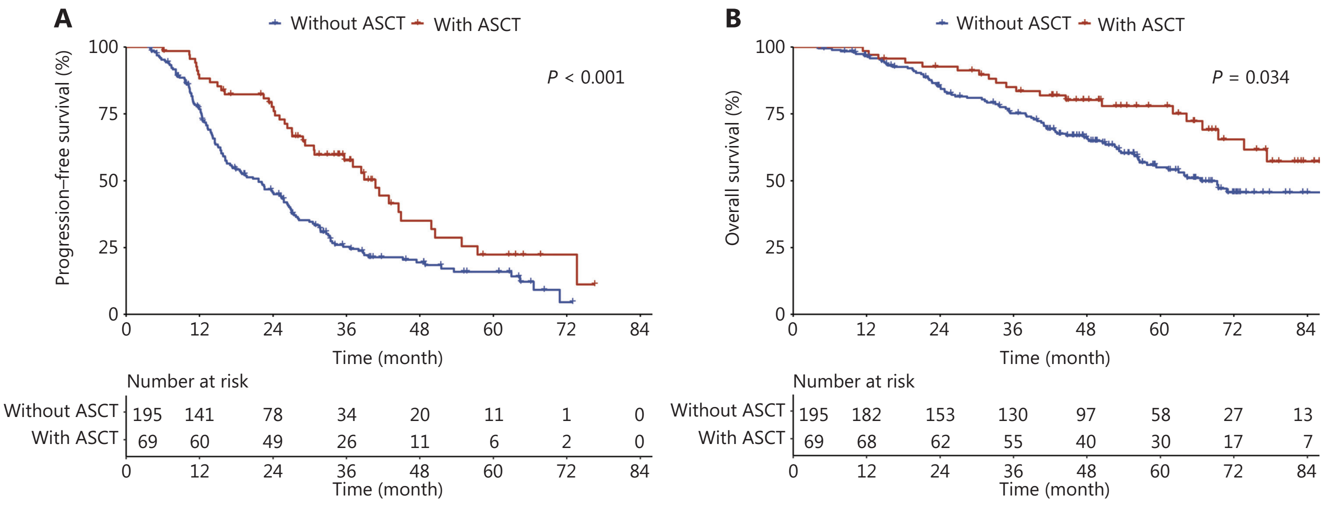

Figure S6 Survival outcomes according to ASCT status among patients with R-ISS II/III disease, including (A) progression-free survival and (B) overall survival by ASCT status among patients with R-ISS II/III disease. ASCT, autologous stem cell transplantation; R-ISS, Revised-International Staging System.

Table S1 Major induction regimens.

PI-based regimens	IMiD-based regimens	PI+IMiD-based regimens	Conventional regimens
VD	RD	VRD	DECP
VC	RCD	VTD	VCR+AD
VAD	TAD	VCDT	VCR+ACD
VCD	TCD	VADR	
VDE	MPT	VMPT	
VDECP	TADC	VADCT	

DECP, dexamethasone/etoposide/cyclophosphamide/cisplatin; IMiD, immunomodulatory drug; MPT, melphalan/prednisolone/thalidomide; PI, proteasome inhibitor; RCD, lenalidomide/cyclophosphamide/dexamethasone; RD, lenalidomide/dexamethasone; TAD, thalidomide/adriamycin/dexamethasone; TADC, thalidomide/adriamycin/dexamethasone/cyclophosphamide; TCD, thalidomide/cyclophosphamide/dexamethasone; VAD, bortezomib/adriamycin/dexamethasone; VADCT, bortezomib/adriamycin/dexamethasone/cyclophosphamide/thalidomide; VADR, bortezomib/adriamycin/dexamethasone/lenalidomide; VC, bortezomib/cyclophosphamide; VCD, bortezomib/cyclophosphamide/dexamethasone; VCDT, bortezomib/cyclophosphamide/dexamethasone/thalidomide; VD, bortezomib/dexamethasone; VDE, bortezomib/dexamethasone/etoposide; VDECP, bortezomib/dexamethasone/etoposide/cyclophosphamide/cisplatin; VCR+AD, vinblastine/adriamycin/dexamethasone; VCR+ACD, vinblastine/adriamycin/cyclophosphamide dexamethasone; VMPT, bortezomib/melphalan/prednisolone/thalidomide; VRD, bortezomib/lenalidomide/dexamethasone; VTD, bortezomib/thalidomide/dexamethasone
